# Results from a test‐and‐treat study for influenza among residents of homeless shelters in King County, WA: A stepped‐wedge cluster‐randomized trial

**DOI:** 10.1111/irv.13092

**Published:** 2023-01-07

**Authors:** Julia H. Rogers, Amanda M. Casto, Gift Nwanne, Amy C. Link, Miguel A. Martinez, Callista Nackviseth, Caitlin R. Wolf, James P. Hughes, Janet A. Englund, Nancy Sugg, Timothy M. Uyeki, Peter D. Han, Brian Pfau, Jay Shendure, Helen Y. Chu

**Affiliations:** ^1^ Division of Allergy and Infectious Diseases, Department of Medicine University of Washington Seattle Washington USA; ^2^ Department of Epidemiology University of Washington Seattle Washington USA; ^3^ Vaccine and Infectious Disease Division Fred Hutchinson Cancer Research Center Seattle Washington USA; ^4^ Department of Biostatistics University of Washington Seattle Washington USA; ^5^ Division of Pediatric Infectious Diseases, Department of Pediatrics University of Washington, Seattle Children's Research Institute Seattle Washington USA; ^6^ Department of Medicine University of Washington Seattle Washington USA; ^7^ Influenza Division, National Center for Immunization and Respiratory Diseases Centers for Disease Control and Prevention Atlanta Georgia USA; ^8^ Brotman Baty Institute for Precision Medicine Seattle Washington USA; ^9^ Department of Genome Sciences University of Washington Seattle Washington USA

**Keywords:** antiviral treatment, homeless shelters, influenza, randomized control trial, rapid molecular influenza test

## Abstract

**Background:**

Persons experiencing homelessness face increased risk of influenza as overcrowding in congregate shelters can facilitate influenza virus spread. Data regarding on‐site influenza testing and antiviral treatment within homeless shelters remain limited.

**Methods:**

We conducted a cluster‐randomized stepped‐wedge trial of point‐of‐care molecular influenza testing coupled with antiviral treatment with baloxavir or oseltamivir in residents of 14 homeless shelters in Seattle, WA, USA. Residents ≥3 months with cough or ≥2 acute respiratory illness (ARI) symptoms and onset <7 days were eligible. In control periods, mid‐nasal swabs were tested for influenza by reverse transcription polymerase chain reaction (RT‐PCR). The intervention period included on‐site rapid molecular influenza testing and antiviral treatment for influenza‐positives if symptom onset was <48 h. The primary endpoint was monthly influenza virus infections in the control versus intervention periods. Influenza whole genome sequencing was performed to assess transmission and antiviral resistance.

**Results:**

During 11/15/2019–4/30/2020 and 11/2/2020–4/30/2021, 1283 ARI encounters from 668 participants were observed. Influenza virus was detected in 51 (4%) specimens using RT‐PCR (A = 14; B = 37); 21 influenza virus infections were detected from 269 (8%) intervention‐eligible encounters by rapid molecular testing and received antiviral treatment. Thirty‐seven percent of ARI‐participant encounters reported symptom onset < 48 h. The intervention had no effect on influenza virus transmission (adjusted relative risk 1.73, 95% confidence interval [CI] 0.50–6.00). Of 23 influenza genomes, 86% of A(H1N1)pdm09 and 81% of B/Victoria sequences were closely related.

**Conclusion:**

Our findings suggest feasibility of influenza test‐and‐treat strategies in shelters. Additional studies would help discern an intervention effect during periods of increased influenza activity.

## 1 BACKGROUND

Seasonal influenza is estimated to have caused between 9–41 million illnesses, 140,710,000 hospitalizations and 12,000–52,000 deaths annually between 2010 and 2020 in the United States.^1^ People experiencing homelessness (PEH) are at risk of severe influenza‐related disease due to high prevalence of underlying conditions, poorly managed substance use disorders, and mental illnesses.^2^ Higher influenza‐related hospitalization and mechanical ventilation rates among PEH compared with housed populations have been observed.^3^

Nearly a third of PEH in the United States stay in emergency shelters or transitional housing programs.^4^ Congregate shelter environments increase transmission risk of influenza and other viral respiratory infections due to poor ventilation, overcrowding, and resident turnover.^5^ Past studies have found a high prevalence of respiratory viruses in shelters, including influenza.^6^, ^7^

Rapid point‐of‐care influenza molecular tests have high sensitivity and specificity.^8^ Within the context of the coronavirus disease 2019 (COVID‐19) pandemic, rapid site‐based tests have been used in shelter settings. These have the advantage of lower cost and faster turnaround time and require less specialized training compared with laboratory‐based molecular testing. Assessments of incident influenza virus infections, the utility of rapid influenza molecular tests in a low‐resource high‐density community setting, and the impact of pharmacologic strategies in sheltered homeless populations remain limited. In this study, we assessed whether point‐of‐care molecular testing and antiviral treatment of influenza was feasible, and whether it reduced influenza transmission in shelters, as compared with no intervention.

## 2 METHODS

### 2.1 Study design overview

We conducted a cluster‐randomized stepped‐wedge trial of a point‐of‐care molecular influenza testing with antiviral treatment intervention in shelters in King County, Washington (WA). The objective of the trial was to evaluate the feasibility and impact of the intervention on the number of secondary influenza cases within homeless shelters. Ethics approval was obtained from the University of Washington Human Subjects Division. The full protocol has been previously described.^9^

### 2.2 Setting and participants

This study was conducted initially at nine homeless shelters in King County, WA. Participants were enrolled over a cumulative 12 months, composed of two 6‐month influenza seasons, from 11/15/19 to 4/30/20 in Year 1 and from 11/2/20 to 4/30/21 in Year 2.

Study eligibility criteria included being a resident at a participating shelter; age ≥ 3 months old; and experiencing new or worsening cough alone or two or more acute respiratory illness (ARI) symptoms in the last 7 days. During the intervention period, criteria included willingness to perform a rapid influenza molecular test and take study medication if the result was positive.

### 2.3 Randomization and intervention

**FIGURE 1:**
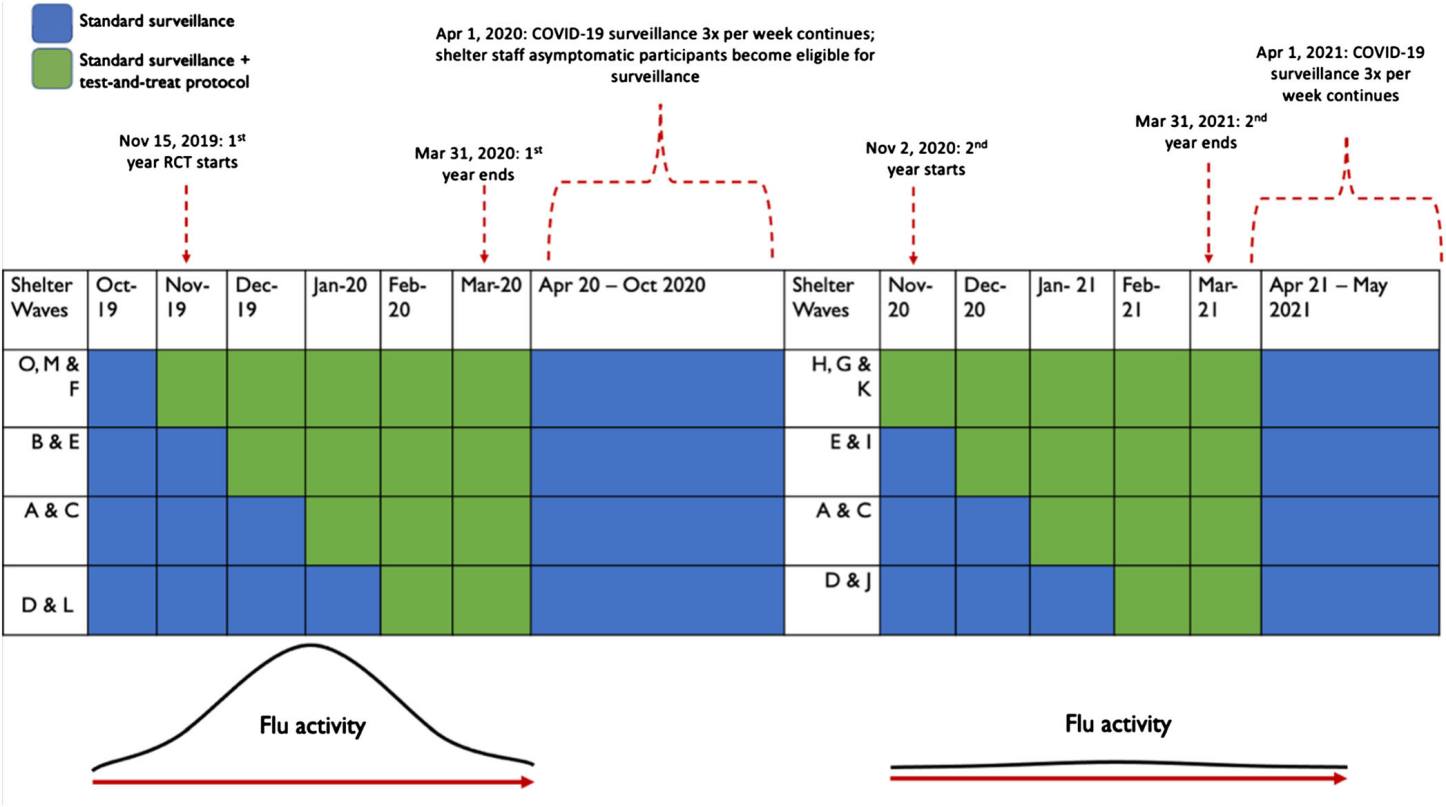
Stepped‐wedge cluster‐randomized trial design and shelter randomization assignments, Years 1 and 2. COVID‐19, coronavirus disease 2019; RCT, randomized controlled trial

We used a stepped‐wedge cluster‐randomized trial, where randomization occurred at the cluster (shelter) level. The design involved monthly random and sequential crossover of clusters from control periods to intervention periods with influenza testing at kiosks until all clusters implemented the intervention. Nine shelters were randomized to the four sequences, with rerandomization at the start of each year (Figure 1) using computer‐generated randomization. Stratified randomization (youth vs. adult shelters) was performed to ensure that the family shelters (n = 3) were evenly distributed to three of the four sequences. All sites remained in the intervention condition for the remainder of the season once it had been introduced.

### 2.4 Study procedures

#### 2.4.1 Recruitment

Participants were recruited from staffed influenza‐surveillance kiosks at each shelter and screened for eligibility. Participants were recruited 6 days per week. To encourage participation, regular staffed kiosk hours were advertised with flyers and regular announcements at shelters. Telephonic translation services were available for participants who did not speak English.

#### 2.4.2 Control period

Eligible individuals had mid‐turbinate nasal swabs collected (self‐collected by participants from 3/6/2020 onwards) using sterile nylon flocked swabs (Copan Diagnostics) and filled surveys providing self‐reported demographic and clinical data on an electric tablet; survey variables and shelter site data have been previously described.^10^ All swabs were sent to a University of Washington (UW) laboratory for reverse transcription polymerase chain reaction (RT‐PCR) testing. No rapid influenza molecular testing or antiviral treatment was offered at the influenza‐surveillance kiosks during the control period.

#### 2.4.3 Intervention period

Trained kiosk staff conducted on‐site rapid molecular influenza testing (Abbott ID NOW, Abbott Laboratories, Lake Bluff, IL, USA), which detects and distinguishes between influenza A and B, and produces a result in 12 min, using nasal specimens collected from participants. Baloxavir (XOFLUZA, Genentech, San Francisco, CA, USA) was administered to all influenza‐positive participants aged ≥12 years. Study clinicians were available by phone to respond to questions or concerns that could not be directly addressed by the kiosk staff. For individuals who tested positive aged 3 months to 11 years, pregnant or breastfeeding, or adults with active malignancy, liver disease, or immunocompromised, a 5‐day treatment course of oseltamivir (TAMIFLU, Roche, Basel, Switzerland) was dispensed. All other individuals aged ≥12 years received a single dose of baloxavir (Appendix S1).

Participants with symptom onset < 48 h were eligible for the intervention as initiation of antiviral treatment is recommended within 48 h of influenza symptom onset for greatest clinical benefits.^11^ Exclusion criteria for the intervention included renal dysfunction; receipt of an antiviral in the past 7 days; and known allergies to baloxavir or oseltamivir. Eligible individuals who had symptom onset > 48 h before enrollment continued to be eligible for surveillance testing, as was available during the control period. Participants who received the intervention also provided an additional nasal swab that was transported to the UW laboratory and subsequently tested for influenza A/B utilizing RT‐PCR.

#### 2.4.4 Follow‐up

**FIGURE 2:**
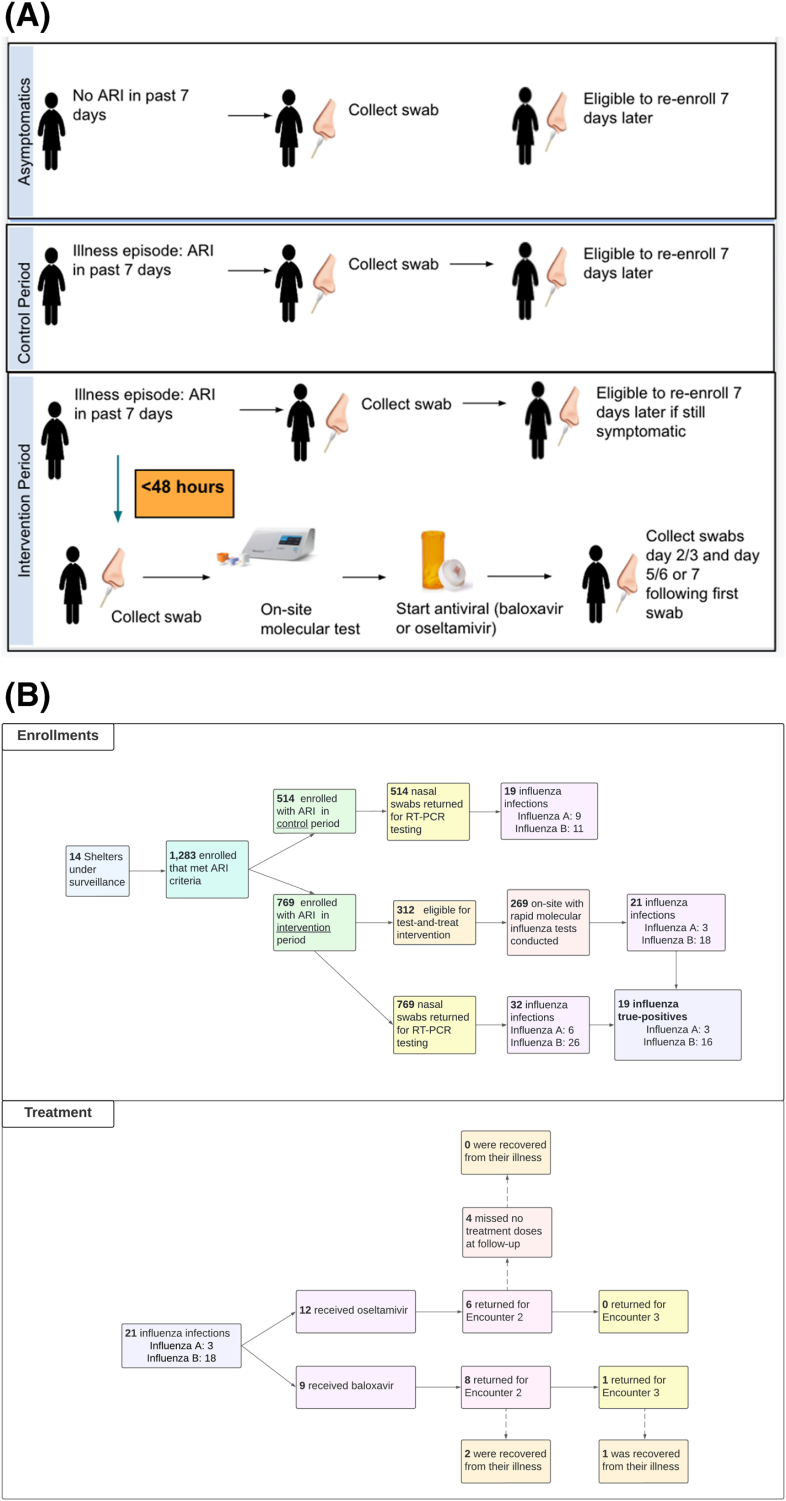
(A) Study design overview including participant‐level study flow of the test‐and‐treat strategy from 11/15/19 to 4/30/21. (B) Total number of participants completing intervention study procedure steps based on eligibility screening. ARI, acute respiratory illness; RT‐PCT, reverse transcription polymerase chain reaction

Following antiviral receipt, influenza‐positive participants were asked to return to the kiosk for symptom surveys and nasal swab collection 2–3 and 5–7 days after diagnosis (Figure 2A). Follow‐up study visit participation was encouraged through autogenerated text‐message reminders for those with cell phones and through paper‐based appointment slips provided by kiosk study staff at time of diagnosis.

### 2.5 Impact of the COVID‐19 pandemic on study protocol

In response to SARS‐CoV‐2 community transmission in WA, study Year 1's intervention was paused on 4/1/2020. Study Year 2 recommenced on 11/2/20 but was terminated early on 4/1/2021 due to operational futility based on minimal influenza activity in King County, WA. During the second year of the study, 5 of the 9 shelters relocated their residents to new facilities to enable improved adherence to SARS‐CoV‐2 transmission mitigation measures (Table S1); in total, 14 shelters were study sites. These new facilities overall had smaller maximum capacities than those used to calculate anticipated study power and estimated sample size (Appendix S1).

Concurrent study recruitment of shelter residents that did not fit ARI criteria to improve SARS‐CoV‐2 surveillance sensitivity was initiated on 4/1/2020. For protocol details and data on non‐ARI influenza virus detection, see Appendix S1.

### 2.6 UW laboratory testing

For samples that were sent to the laboratory, total nucleic acids were extracted using the Magna Pure 96 kit (Roche) and tested by TaqMan OpenArray RT‐PCR (Thermo) for multiple viral pathogens, including influenza A (H3 and H1 hemagglutinin subtypes) and influenza B. OpenArray relative threshold (CRT) values were used to determine the viral load of each sample; CRT values are inversely related to the viral load. Viral genome sequencing by hybrid capture was attempted on all influenza‐positive samples with viral loads > ~50,000 genomic copies/ml using a protocol described previously.^12^

### 2.7 Outcomes and statistical analyses

All data in this analysis are presented by participant encounter, defined as each time an eligible individual provided a nasal swab and completed a survey with study staff. We used descriptive statistics to evaluate the sociodemographic and clinical characteristics at baseline in control and intervention periods for study Years 1 and 2; these characteristics were also described by lab‐confirmed influenza result. Descriptive and statistical analyses were performed using R Statistical Software (Version 4.0.3, Foundation for Statistical Computing, Vienna, Austria).

#### 2.7.1 Primary objective: Effects on reducing secondary spread of influenza virus

The primary endpoint was monthly number of influenza‐positive samples in the control versus intervention periods among ARI‐participant encounters. The predictor of interest was test‐and‐treat at the shelter/cluster‐month level (i.e., intervention vs. control periods). Shelters were analyzed with an intent‐to‐treat (ITT) analysis to preserve the advantages of randomization. Relative risk of influenza virus infection during the intervention period compared with the control period was calculated using a generalized linear mixed model (GLMM) following a Poisson distribution with a log link. The model was adjusted for calendar time with an offset of shelter maximum capacity and random effect for shelter and time. Due to low influenza virus circulation in Year 2, the primary outcome analysis for this study was calculated based on data collected only from Year 1. Influenza‐like illness (ILI) was defined as fever and cough, or fever and sore throat.

#### 2.7.2 Secondary objective A: Assess feasibility of test‐and‐treat strategy for influenza in shelters

Feasibility of implementation of point‐of‐care influenza molecular testing was measured as the time between symptom onset until diagnosis through rapid test or laboratory test. Feasibility of implementation of antiviral treatment was measured as the time between symptom onset until initiation of antiviral treatment. Additional endpoints used to characterize feasibility were proportion of participants lost‐to‐follow‐up (LTFU) and proportion of participants non‐compliant with oseltamivir therapy (self‐reported measure collected during on‐site follow‐up visits with kiosk staff).

#### 2.7.3 Secondary objective B: Characterize influenza transmission

To better understand the relationship among the influenza cases detected by this study and between these cases and cases with a viral genomic sequence publicly available in the GISAID database, we attempted sequencing on all influenza‐positive samples from Year 1 with viral loads > ~50,000 genomic copies/ml. We were able to generate influenza genome sequences for 23 of these samples (7 influenza A(H1N1)pdm09 and 16 influenza B/Victoria) (Table S3). Influenza A(H1N1)pdm09 and influenza B phylogenetic trees including these genomes and influenza genomes in GISAID from samples collected in WA during the study period (10/2019–3/2020) were created and pairwise genetic (Hamming) distances were calculated for all influenza A/B sequence pairs. We also assessed the viral genomes generated for the study for known mutations associated with reduced susceptibility to baloxavir and oseltamivir in persons during and following antiviral treatment.

## 3 RESULTS

### 3.1 Study population and demographics

Overall, we observed 1283 ARI encounters (1159 from Year 1 and 124 from Year 2) from 668 unique participants who met eligibility criteria. Of these, 514 and 769 encounters occurred during the control and intervention periods, respectively. The median age of participants was 45 years (interquartile range [IQR]: 24), and 8.3% were children (Table 1). The study population predominantly identified as male (69.1%), White (52.0%), and non‐Hispanic/Latinx (88.9%). A majority of encounters were from current smokers (63.4%), and nearly all had health insurance (90.5%); a minority reported receiving the current season's influenza vaccine (41.1%). Most participants used shelters as their usual nighttime accommodation (73.3%).

**TABLE 1:** Baseline sociodemographic and health characteristics of ARI‐participant encounters, overall and by study period, 2019–2020 and 2020–2021 influenza seasons

	Study period		
	Control (n = 514)	Intervention (n = 769)	Overall participant encounters (N = 1283)
***Sociodemographic variables***			
Age, median [IQR]	42.0 [24.0]	49.0 [24.0]	45.0 [24.0]
Age group			
3 months to 4 years	25 (4.86%)	37 (4.81%)	62 (4.83%)
5–11 years	14 (2.72%)	15 (1.95%)	29 (2.26%)
12–17 years	7 (1.36%)	8 (1.04%)	15 (1.17%)
18–49 years	301 (58.6%)	330 (42.9%)	631 (49.2%)
50–64 years	145 (28.2%)	340 (44.2%)	485 (37.8%)
≥65 years	22 (4.28%)	39 (5.07%)	61 (4.75%)
Male sex	298 (58.5%)	580 (76.2%)	878 (69.1%)
Race			
American Indian and Alaskan Native	13 (2.80%)	25 (3.71%)	38 (3.34%)
Asian	11 (2.37%)	12 (1.78%)	23 (2.02%)
Black/African American	127 (27.3%)	192 (28.5%)	319 (28.0%)
Multiple	65 (14.0%)	84 (12.5%)	149 (13.1%)
Native Hawaiian and Pacific Islander	15 (3.23%)	2 (0.297%)	17 (1.49%)
White	234 (50.3%)	358 (53.2%)	592 (52.0%)
Hispanic/Latinx ethnicity	69 (13.6%)	71 (9.37%)	140 (11.1%)
Employed	80 (17.0%)	152 (21.4%)	232 (19.6%)
Education			
Less than high school	85 (18.4%)	113 (16.1%)	198 (17.0%)
High school or GED	192 (41.6%)	291 (41.5%)	483 (41.5%)
Some college	131 (28.4%)	223 (31.8%)	354 (30.4%)
Bachelor's degree or higher	53 (11.5%)	75 (10.7%)	128 (11.0%)
***Health variables***			
Has health insurance	481 (94.3%)	665 (88.0%)	1146 (90.5%)
Received current season's influenza vaccine (self‐report)	198 (40.9%)	305 (41.2%)	503 (41.1%)
Current smoker	345 (67.1%)	469 (61.0%)	814 (63.4%)
Pregnant	12 (6.28%)	7 (4.40%)	19 (5.43%)
**Lifestyle variables**			
Sleeping location			
Communal	395 (76.8%)	618 (80.4%)	1013 (79.0%)
Cubicles	10 (1.95%)	15 (1.95%)	25 (1.95%)
Private/family room	109 (21.2%)	136 (17.7%)	245 (19.1%)
Duration of homelessness			
<6 months	177 (35.2%)	221 (29.7%)	398 (31.9%)
6–12 months	97 (19.3%)	111 (14.9%)	208 (16.7%)
13–24 months	58 (11.5%)	90 (12.1%)	148 (11.9%)
>24 months	171 (34.0%)	322 (43.3%)	493 (39.5%)
Usual nighttime accommodations^a^			
Shelter	377 (73.3%)	563 (73.3%)	940 (73.3%)
Transitional housing	13 (2.53%)	22 (2.86%)	35 (2.73%)
Street/outside/tent/encampment	68 (13.2%)	89 (11.6%)	157 (12.2%)
Abandoned building/squat	5 (0.973%)	3 (0.391%)	8 (0.624%)
Vehicle	19 (3.70%)	37 (4.82%)	56 (4.37%)
Hotel or motel	25 (4.86%)	46 (5.99%)	71 (5.54%)
Other	4 (0.778%)	4 (0.521%)	8 (0.624%)
Unknown	3 (0.584%)	4 (0.521%)	7 (0.546%)

*Note*: All columns apart from “Total” have calculated row percentages; “Total” column percentages calculated exclude missing responses.

Abbreviations: ARI, acute respiratory illness; IQR, interquartile range.

^a^ Not mutually exclusive.

### 3.2 Clinical characteristics of ARI encounters

The most common symptoms reported were rhinorrhea (76.9%) and cough (72.3%) (Table 2). The most common comorbidities were chronic lung disease (24.9%) and diabetes (14%). The proportion of ARI encounters that met the ILI case definition was 26.3%. The majority reported symptom onset within 5–7 days (37.7%); however, only 23.9% reported having sought medical care for their illness episode (25.5% of ARI encounters that resulted in medical care‐seeking were influenza‐positive), and 14 (4.6%) of those with ARI that received care were prescribed an antiviral.

**TABLE 2:** Baseline sociodemographic and clinical characteristics of ARI‐participant encounters, by laboratory multiplex assay‐confirmed influenza result and rapid influenza molecular test result, 2019–2020 and 2020–2021 influenza seasons

Variable	All lab‐confirmed influenza results			All rapid‐test‐confirmed influenza results		
	Positive (n = 51)	Negative (n = 1232)	Overall (N = 1283)	Positive (n = 21)	Negative (n = 248)	Overall (N = 269)
Age, median [IQR]	28.0 [34.0]	46.0 [25.0]	45.0 [24.0]	19.0 [26.0]	45.0 [27.0]	43.0 [28.0]
Age group						
3 months to 4 years	9 (17.6%)	53 (4.30%)	62 (4.83%)	8 (38.1%)	13 (5.24%)	21 (7.81%)
5–11 years	5 (9.80%)	24 (1.95%)	29 (2.26%)	1 (4.76%)	11 (4.44%)	12 (4.46%)
12–17 years	1 (1.96%)	14 (1.14%)	15 (1.17%)	1 (4.76%)	5 (2.02%)	6 (2.23%)
18–49 years	28 (54.9%)	603 (48.9%)	631 (49.2%)	7 (33.3%)	134 (54.0%)	141 (52.4%)
50–64 years	6 (11.8%)	479 (38.9%)	485 (37.8%)	4 (19.0%)	80 (32.3%)	84 (31.2%)
≥65 years	2 (3.92%)	59 (4.79%)	61 (4.75%)	0 (0%)	5 (2.02%)	5 (1.86%)
Male sex	31 (60.8%)	847 (69.5%)	878 (69.1%)	10 (47.6%)	159 (65.2%)	169 (63.8%)
Race						
American Indian and Alaskan Native	1 (2.00%)	37 (3.40%)	38 (3.34%)	0 (0%)	6 (2.74%)	6 (2.50%)
Asian	0 (0%)	23 (2.11%)	23 (2.02%)	0 (0%)	4 (1.83%)	4 (1.67%)
Black/African American	19 (38.0%)	300 (27.6%)	319 (28.0%)	8 (38.1%)	56 (25.6%)	64 (26.7%)
Multiple	3 (6.00%)	146 (13.4%)	149 (13.1%)	2 (9.52%)	14 (6.39%)	16 (6.67%)
Native Hawaiian and Pacific Islander	0 (0%)	17 (1.56%)	17 (1.49%)	0	0	0
White	27 (54.0%)	565 (51.9%)	592 (52.0%)	11 (52.4%)	139 (63.5%)	150 (62.5%)
Comorbidities						
Blood disorders (e.g., sickle cell)	0 (0%)	26 (2.12%)	26 (2.03%)	0 (0%)	7 (2.82%)	7 (2.60%)
Chronic lung disease^a^	13 (23.6%)	306 (24.9%)	319 (24.9%)	2 (9.52%)	52 (21.0%)	54 (20.1%)
Cancer/immunosuppression (by medication or disease)	0 (0%)	50 (4.07%)	50 (3.90%)	0 (0%)	13 (5.24%)	13 (4.83%)
Diabetes	14 (25.5%)	165 (13.4%)	179 (14.0%)	1 (4.76%)	16 (6.45%)	17 (6.32%)
Heart disease (heart failure or heart attack)	9 (16.4%)	62 (5.05%)	71 (5.53%)	1 (4.76%)	6 (2.42%)	7 (2.60%)
Liver or kidney disease	0 (0%)	74 (6.03%)	74 (5.77%)	0 (0%)	17 (6.85%)	17 (6.32%)
Influenza‐like illness (ILI)^b^	23 (45.1%)	315 (25.6%)	338 (26.3%)	11 (52.4%)	65 (26.2%)	76 (28.3%)
Symptoms						
Feeling feverish	27 (52.9%)	386 (31.3%)	413 (32.2%)	12 (57.1%)	92 (37.1%)	104 (38.7%)
Cough	42 (82.4%)	885 (71.8%)	927 (72.3%)	18 (85.7%)	162 (65.3%)	180 (66.9%)
Rhinorrhea	41 (80.4%)	945 (76.7%)	986 (76.9%)	19 (90.5%)	198 (79.8%)	217 (80.7%)
Chills	17 (33.3%)	362 (29.4%)	379 (29.5%)	6 (28.6%)	72 (29.0%)	78 (29.0%)
Sweats	15 (29.4%)	346 (28.1%)	361 (28.1%)	7 (33.3%)	65 (26.2%)	72 (26.8%)
Sore throat	17 (33.3%)	502 (40.7%)	519 (40.5%)	8 (38.1%)	109 (44.0%)	117 (43.5%)
Nausea or vomiting	20 (39.2%)	330 (26.8%)	350 (27.3%)	5 (23.8%)	67 (27.0%)	72 (26.8%)
Headache	18 (35.3%)	507 (41.2%)	525 (40.9%)	6 (28.6%)	117 (47.2%)	123 (45.7%)
Fatigue	23 (45.1%)	551 (44.7%)	574 (44.7%)	12 (57.1%)	122 (49.2%)	134 (49.8%)
Myalgia	21 (41.2%)	550 (44.6%)	571 (44.5%)	8 (38.1%)	114 (46.0%)	122 (45.4%)
Increased trouble breathing	11 (21.6%)	278 (22.6%)	289 (22.5%)	4 (19.0%)	66 (26.6%)	70 (26.0%)
Diarrhea^c^	13 (25.5%)	210 (17.0%)	223 (17.4%)	3 (14.3%)	40 (16.1%)	43 (16.0%)
Ear pain or ear discharge^c^	3 (5.88%)	130 (10.6%)	133 (10.4%)	1 (4.76%)	19 (7.66%)	20 (7.43%)
Symptom onset < 48 h	32 (64.7%)	447 (36.3%)	480 (37.4%)	21 (100%)	148 (100%)	169 (100%)
Sought clinical care for illness episode	13 (25.5%)	293 (23.8%)	306 (23.9%)	1 (4.76%)	25 (10.1%)	26 (9.67%)
Received an antiviral from a clinical provider for illness episode (n = 306)^d^						
Yes	4 (30.8)	10 (3.41%)	14 (4.58%)	NA	NA	NA
No	8 (61.5%)	273 (93.2%)	281 (91.8%)	NA	NA	NA
Do not know	1 (7.69%)	10 (3.41%)	11 (3.59%)	NA	NA	NA

Abbreviations: ARI, acute respiratory illness; IQR, interquartile range; NA, not applicable.

^a^ Chronic obstructive pulmonary disorder, emphysema, asthma, or reactive airway disease.

^b^ Fever and cough or fever and sore throat.

^c^ Only eligible trigger symptoms for participants < 18 years.

^d^ Not applicable to participants who had a rapid test conducted as prior antiviral treatment was an exclusion criterion for receipt of the intervention.

### 3.3 Influenza detection and intervention effect on reducing secondary spread of influenza virus

Among all ARI‐participant encounters, 51 (4.0%) influenza virus infections (A = 15; B = 37) were identified through RT‐PCR. Of the influenza A subtypes, A(H1N1)pdm09 predominated (93.3%). Most infections were identified among participants 18–59 years (54.9%; Table 2); 17.6% were among children 3 months to 5 years. No influenza virus infections were detected by RT‐PCR in study Year 2; one was detected by rapid influenza molecular test.

Among the 269 ARI encounters that were eligible for the intervention, 21 (7.8%; Table 2) were influenza‐positive by rapid influenza molecular test. Most infections detected by rapid molecular test were identified among participants aged 3 months to 5 years (38.1%). See Appendix S1 for RT‐PCR and rapid molecular test concordance results.

Overall, more infections were identified during intervention periods (n = 32) compared with control periods (n = 19). Restricting analysis to Year 1 of the study, the relative risk of infection during the intervention periods compared with control periods, adjusted for calendar time, was 1.73 (95% confidence interval [CI] 0.50–6.00, p‐value = 0.386; Table 3). Although these results were not statistically significant, we did observe that family shelters had significantly higher test positivity compared with adult‐only shelters (11% vs. 2%; p < 0.001) across both study years.

**TABLE 3:** Relative risk of infection during the intervention period compared with the control period using a generalized linear mixed model following a Poisson distribution with a log link and robust variance, adjusted for calendar time and an exposure time variable based on shelter capacity. This model includes ARI‐participant encounters from Year 1 of the study (11/15/2019–4/31/2020).

Shelter names		Randomized waves					
		Nov‐19	Dec‐19	Jan‐20	Feb‐20	Mar‐20	Apr‐20
O, M, and F	Influenza cases	6	1	5	1	0	0
	Persons at risk^a^	372	372	372	372	372	372
B and E	Influenza cases	1	14	1	0	0	0
	Persons at risk	170	170	170	170	170	170
A and C	Influenza cases	1	0	2	0	0	0
	Persons at risk	105	105	105	105	105	105
D and L	Influenza cases	1	3	13	2	0	0
	Persons at risk	385	385	385	385	385	385

			**Risk ratio**	**95% confidence interval**		**p‐value**	
Influenza virus infection determined by RT‐PCR			1.73	0.50–6.00		0.386	

*Note*: 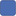, Standard surveillance; 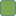, standard surveillance + test‐and‐treat protocol.

Abbreviations: ARI, acute respiratory illness; RT‐PCR, reverse transcription polymerase chain reaction.

^a^ Persons at risk determined by static measure of maximum nightly shelter capacity.

### 3.4 Feasibility of influenza test‐and‐treat strategy

Of all ARI‐participant encounters, 37.4% reported symptom onset < 48 h (Table 2; Figure 2B). Among the 769 ARI encounters observed at shelters during intervention periods, 312 were eligible for, and 269 (86.2%) completed, rapid influenza molecular testing; 21 (7.8%) of these were influenza‐positive (A = 3; B = 18).

All 21 positives were treated with an antiviral, including 12 with oseltamivir and 9 with baloxavir; 38% of those treated were <5 years old. Of the 51 symptomatic infections identified through RT‐PCR, 64.7% had symptom onset < 48 h of specimen collection. Of the 32 symptomatic infections identified through RT‐PCR during intervention periods, 19 (59.4%) also were detected <48 h of symptom onset by the rapid molecular test and received immediate antiviral treatment. Of the 6 oseltamivir recipients with follow‐up data, 4 (66%) were fully treatment adherent with no missed doses. Of the 21 participants who received either antiviral, 14 (66.7%) returned for their first follow‐up study visit; only 1 (4.8%) returned for both study visits in the week following treatment. No severe adverse events were identified over the study period.

### 3.5 Genomics

**FIGURE 3:**
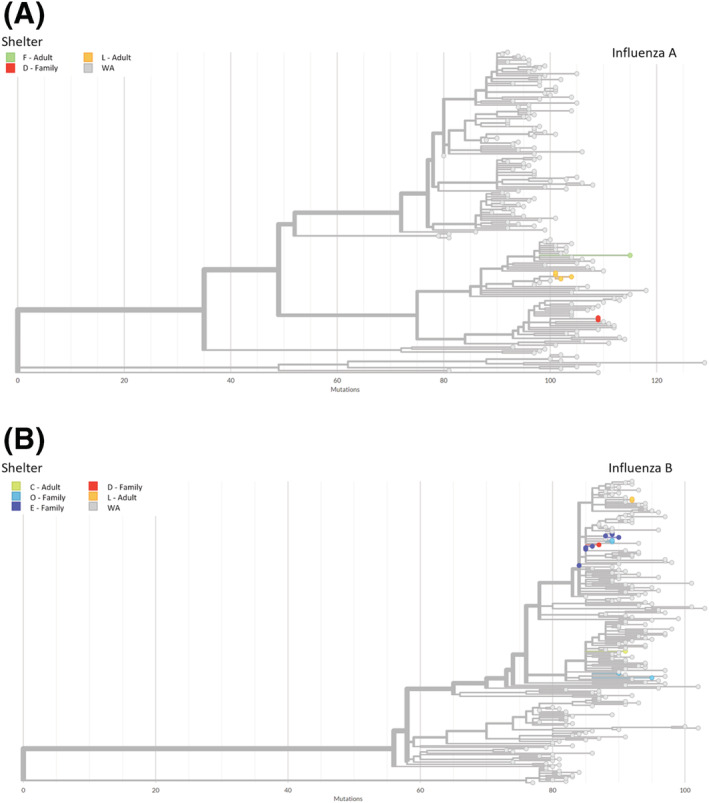
Maximum likelihood phylogenetic trees for (A) influenza A and (B) influenza B. Trees include all sequenced study samples and all genomes for samples collected in Washington (WA) during the study timeframe that have been deposited in GISAID.

All seven full genome sequences generated for the influenza A‐positive samples from three different shelters were identified as A(H1N1)pdm09 viruses. We generated a maximum likelihood phylogenetic tree containing these seven samples along with all A(H1N1)pdm09 genomes deposited in GISAID that were collected in WA from 10/2019 to 3/2020 (N = 158) (Figure 3A). Two clusters of multiple shelter samples were identified. All four sequenced A(H1N1)pdm09 samples from shelter L grouped together in the tree with 99% bootstrap support; two of these samples had identical sequences. The two sequenced A(H1N1)pdm09 samples from shelter D were also identical in sequence. We estimated that at least four out of seven sequenced influenza A samples represented a case resulting from intra‐shelter transmission.

All 16 full genome sequences generated for the influenza B‐positive samples from five different shelters were identified as Victoria lineage viruses. A phylogenetic tree containing these samples along with all Victoria genomes deposited in WA GISAID collected from 10/2019 to 3/2020 (N = 189) was generated (Figure 3B). Genetic diversity was observed in several clusters of multiple shelter samples. Four out of eight sequenced samples from family shelter E grouped together (two of these were identical) with bootstrap support of 100%; two of the remaining shelter E sequences were also identical to each other. In addition, two out of four samples from family shelter O clustered together (100% bootstrap support) as did two out of two samples from shelter L (100% bootstrap support). We estimated that at least 5 out of 16 sequenced influenza B samples represented a case resulting from intra‐shelter transmission.

A total of 86% (6 of 7) of sequenced A(H1N1)pdm09 and 81% (13 of 16) of B/Victoria lineage viruses were most closely related to another sequence from the same shelter when analyzed with WA GISAID sequences. There were cases where inter‐shelter transmission was a possibility, most notably the close relationship between the influenza B shelter D sample and two shelter E samples (pairwise distance of two). The average pairwise distances between influenza A genomes and influenza B genomes from the same shelter were 2.0 and 7.0, respectively, versus average pairwise distances of 53.4 and 16.8 for genomes from different shelters.

The NA and PA genic regions of the 23 shelter genomes were reviewed at the consensus level for known mutations associated with reduced susceptibility to oseltamivir or baloxavir, respectively, and no evidence of reduced antiviral susceptibility was identified.

## 4 DISCUSSION

This study assessed the feasibility and impact of an on‐site test‐and‐treat intervention for influenza among persons experiencing homelessness in a congregate setting. Although the study was limited by operational futility from a near absence of influenza virus circulation in WA in Year 2,^13^ we found that use of a rapid molecular point‐of‐care test‐and‐treat strategy for influenza at shelters was feasible, whereas the intervention had no significant effect on influenza incidence.

Using on‐site surveillance, we observed a substantial proportion of overall ARI encounters (37.4%) within 48 h, a group that would be eligible to receive antiviral treatment if influenza‐positive. The COVID‐19 pandemic has also shown that rapid viral testing at shelters is feasible and effective when combined with mitigation measures; however, the impact of using antivirals for influenza treatment and chemoprophylaxis to reduce intra‐shelter transmission has not yet been explored.^14^

A majority of RT‐PCR influenza‐positive participants identified during intervention periods received antiviral treatment (59.4%), suggesting that immediate treatment is feasible. Use of single‐dose baloxavir treatment in this study was an advantage as it was compliance independent and has shown to be effective as both a prophylactic and means of reducing secondary influenza transmission in households.^15^, ^16^ However, we also found high acceptability of antiviral treatment among non‐baloxavir eligible participants, despite oseltamivir's more complex 5‐day regimen.

We found that less than half of symptomatic influenza virus infections met ILI criteria. This suggests that the ILI definition is less valuable as a diagnostic criterion than a means of surveilling community‐level influenza virus circulation and that viral diagnostic testing is needed to distinguish signs and symptoms caused by specific viral infections. Based on the World Health Organization (WHO) global influenza update from June 2022, countries are recommended to prepare for the co‐circulation of influenza and SARS‐CoV‐2 viruses and to enhance integrated surveillance to monitor influenza and SARS‐CoV‐2 simultaneously.^17^ Considering the renewed global circulation of influenza A viruses, this study provides a framework to further assess the integration of rapid influenza diagnostic test (RIDT) and access to recommended therapeutics for improved surveillance and response in congregate settings.

In this study, rapid influenza molecular testing had high concordance with RT‐PCR results, supporting use in shelters (see Appendix S1). In clinical settings, RIDT utilization, despite being less sensitive than rapid molecular influenza testing,^18^ has been found to reduce overall influenza‐related health care costs and improve proper utilization of influenza antivirals.^19^ PEH, however, are disproportionately dependent on hospital and emergency services compared with the general population and for influenza‐related illnesses, PEH patients have been found to experience substantially higher rates of hospitalization than non‐homeless patients.^3^, ^20^ During the 2009 H1N1 pandemic, observed hospitalization rates were up to 29 times higher among PEH.^3^ Accessible shelter‐based rapid molecular tests have the potential to significantly improve influenza diagnostic accuracy over less sensitive rapid influenza antigen tests to facilitate prompt antiviral treatment and control measures in a variety of congregate facilities with high risk of influenza outbreaks. This approach also has the potential to save hospital resources and reduce overall costs on the health care system during seasonal influenza epidemics and pandemics.^3^

We found that sequenced influenza viruses from the same shelter were frequently closely related, likely reflective of intra‐shelter transmission. However, there were two examples of genetic diversity among samples collected from a single shelter over a short time period, both from family shelters, raising the possibility of multiple influenza viral introductions.^21^ Although our sample size was small, this raises the question of whether transmission of influenza and other respiratory viruses differs between adult‐only versus family shelters. We also observed that sequenced samples from different shelters were not closely related, which would argue against transmission of influenza between the study shelters.

Individuals frequently sought clinical care for their ARI in this study, yet few were prescribed an antiviral prior to study enrollment. This may be due to lack of provider awareness regarding antiviral treatment, or delays in seeking clinical care outside of the shelter setting making outside the recommended 48‐h window for antiviral treatment since symptom onset.^22^ This is supported by studies reporting that PEH are likely to delay seeking care for acute infections due to multiple barriers (including transportation, provider discrimination, and inaccessibility).^23^, ^24^ Studies have found key enablers to any vaccine uptake among PEH are convenient locations and times, and incorporation of vaccination into routine health and social care.^25^ Acute respiratory illness testing and treatment uptake among sheltered PEH likely require similar enabling environments (e.g., rapid antiviral delivery on‐site).

This study was subject to several limitations. First, the COVID‐19 pandemic and subsequent reduction of influenza virus circulation led to low study power and limited ability to assess the effect of the intervention. We therefore view these results as inconclusive rather than negative. Second, the use of shelter capacity to determine persons at risk in the GLMM calculation does not account for resident transiency and may have over‐estimated the population if shelters were not at maximum capacity during study Year 1. Third, selection biases may have occurred as the nature of the stepped‐wedge cluster‐randomized trial design does not allow for blinding of the intervention. Study participation may have been perceived as more desirable during intervention periods when immediate testing results and actionable intervention for illness episodes were made available. Fourth, our study design may have under‐estimated influenza virus transmission in shelters as we did not assess transmission from residents with asymptomatic infection or capture secondary asymptomatic infections. Finally, survey data were based on self‐report, which may be subjective particularly for variables such as symptoms experienced and illness duration.

## 5 CONCLUSION

Our findings establish the feasibility of an on‐site influenza test‐and‐treat strategy in shelters that has the potential to be applied during influenza epidemics and pandemics. Our genomic data suggest that intra‐shelter spread of influenza viruses is common and is responsible for a large proportion of symptomatic influenza virus infections in shelters. Possible distinct transmission dynamics within family and adult shelters suggests that interventions tailored to shelters serving children should be explored (e.g., on‐site antiviral treatment for symptomatic residents and chemoprophylaxis for exposed residents, baloxavir‐only treatment for children ≥ 5 years old,^26^ or improved ventilation systems and other non‐pharmaceutical interventions). The effect of shelter‐level interventions on mitigating influenza transmission, morbidity, and mortality among PEH should be assessed through additional studies.

## CONFLICTS OF INTEREST

Dr. Chu reported consulting with Ellume, Pfizer, The Bill and Melinda Gates Foundation, GlaxoSmithKline, and Merck. She has received research funding from Sanofi Pasteur, and support and reagents from Ellume and Cepheid outside of the submitted work. Dr. Englund reported research support from Merck, AstraZeneca, Pfizer, and GlaxoSmithKline. She is a consultant for Meissa Vaccines, Sanofi Pasteur, and AstraZeneca.

## AUTHOR CONTRIBUTIONS

**Julia H. Rogers:** Conceptualization; data curation; formal analysis; methodology; project administration; writing‐original draft; writing‐review and editing. **Amanda M. Casto:** Conceptualization; formal analysis; visualization; writing‐original draft. **Gift Nwanne:** Conceptualization; formal analysis; project administration; writing‐original draft; writing‐review and editing. **Amy C. Link:** Project administration; resources; writing‐review and editing. **Miguel A. Martinez:** Formal analysis; project administration; resources; writing‐review and editing. **Callista Nackviseth:** Investigation; project administration; resources; writing‐review and editing. **Caitlin R. Wolf:** Investigation; resources; software; writing‐review and editing. **James P. Hughes:** Conceptualization; formal analysis; investigation; methodology; supervision; writing‐review and editing. **Janet A. Englund:** Conceptualization; formal analysis; investigation; methodology; supervision; writing‐review and editing. **Nancy Sugg:** Conceptualization; methodology; supervision; writing‐review and editing. **Timothy M. Uyeki:** Formal analysis; investigation; methodology; supervision; writing‐review and editing. **Peter D. Han:** Data curation; project administration; resources; software; validation; writing‐review and editing. **Brian Pfau:** Data curation; investigation; project administration; resources; software; writing‐review and editing. **Jay Shendure:** Conceptualization; investigation; methodology; resources; validation; writing‐review and editing. **Helen Y. Chu:** Conceptualization; funding acquisition; investigation; methodology; project administration; resources; supervision; validation; writing‐review and editing.

## ETHICS STATEMENT

The University of Washington Institutional Review Board approved this study. All participants provided informed consent and/or assent.

### PEER REVIEW

The peer review history for this article is available at https://publons.com/publon/10.1111/irv.13092.

## Supporting information


**Table S1.** Shelter‐specific characteristics and ARI participant encounter numbers, 2019–2020 and 2020–2021 influenza seasons
**Table S2.** Rapid on‐site molecular test results in comparison with RT‐PCR‐confirmed influenza test results
**Table S3.** Influenza‐positive specimens with full genome sequences collected from shelter residents with <50% missing data.
**Figure S1.** Weekly influenza virus detection by RT‐PCR, October 2019 – May 2021; includes nasal specimens collected from non‐ARI surveillance concurrently conducted at study site shelters
**Figure S2.** Within‐subject change in viral load of specimen with detectable influenza RNA virus by RT‐PCR at study days 0, 2/3 and days 5/6/7 among those treated with an antiviral

## Data Availability

Patient‐level data and statistical code are available upon request from the corresponding author at jr66@uw.edu.

## References

[1] Disease burden of flu. Centers for Disease Control and Prevention. https://www.cdc.gov/flu/about/burden/index.html. Published 2022. Accessed October 18, 2022.

[2] Hwang SW , Orav EJ , O'Connell JJ , Lebow JM , Brennan TA . Causes of death in homeless adults in Boston. Ann Intern Med. 1997;126(8):625‐628. doi:10.7326/0003-4819-126-8-199704150-00007 9103130

[3] Miyawaki A , Hasegawa K , Tsugawa Y . Lessons from influenza outbreaks for potential impact of COVID‐19 outbreak on hospitalizations, ventilator use, and mortality among homeless persons in New York state. J Gen Intern Med. 2020;35(9):2781‐2783. doi:10.1007/s11606-020-05876-1 32500333 PMC7272140

[4] Henry M , de Sousa T , Roddey C , Gayen S , Bednar TJ . *The 2020 Annual Homeless Assessment Report (AHAR) to Congrress*. 2020. https://www.huduser.gov/portal/sites/default/files/pdf/2020-AHAR-Part-1.pdf

[5] Tsai J , Wilson M . COVID‐19: a potential public health problem for homeless populations. Lancet Public Heal. 2020;5(4):e186‐e187. doi:10.1016/S2468-2667(20)30053-0 PMC7104053 32171054

[6] Ly TDA , Hoang VT , Goumballa N , et al. Variations in respiratory pathogen carriage among a homeless population in a shelter for men in Marseille, France, March–July 2020: cross‐sectional 1‐day surveys. Eur J Clin Microbiol Infect Dis. 2021;40(7):1579‐1582. doi:10.1007/s10096-020-04127-9 33580843 PMC7881748

[7] Mosites E , Parker EM , Clarke KEN , et al. Assessment of SARS‐CoV‐2 infection prevalence in homeless shelters—four U.S. cities, March 27–April 15, 2020. MMWR Morb Mortal Wkly Rep. 2020;69(17):521‐522. doi:10.15585/mmwr.mm6917e1 32352957 PMC7206983

[8] Woodberry MW , Shankar R , Cent A , Jerome KR , Kuypers J . Comparison of the Simplexa FluA/B & RSV direct assay and laboratory‐developed real‐time PCR assays for detection of respiratory virus. J Clin Microbiol. 2013;51(11):3883‐3885. doi:10.1128/JCM.02395-13 24048529 PMC3889790

[9] Newman KL , Rogers JH , McCulloch D , et al. Point‐of‐care molecular testing and antiviral treatment of influenza in residents of homeless shelters in Seattle, WA: study protocol for a stepped‐wedge cluster‐randomized controlled trial. Trials. 2020;21(1):956. doi:10.1186/s13063-020-04871-5 33228787 PMC7682130

[10] Chow EJ , Casto AM , Roychoudhury P , et al. The clinical and genomic epidemiology of rhinovirus in homeless shelters—King County, Washington. J Infect Dis. 2022;226(Supplement_3):S304‐S314. doi:10.1093/infdis/jiac239 35749582 PMC9384451

[11] CDC Centers for Diseases Control and Report . Influenza antiviral medications: summary for clinicians. MMWR, Morbidity & Mortality Weekly Report. http://www.cdc.gov/flu/professionals/antivirals/summary-clinicians.htm. Published 2016. Accessed October 18, 2022.

[12] Chu HY , Boeckh M , Englund JA , et al. The Seattle Flu Study: a multiarm community‐based prospective study protocol for assessing influenza prevalence, transmission and genomic epidemiology. BMJ Open. 2020;10(10):e037295. doi:10.1136/bmjopen-2020-037295 PMC7542952 33033018

[13] *Washington State Influenza Summary: 2020–2021 Season*; 2021.

[14] Zhu A , Bruketa E , Svoboda T , et al. Respiratory infectious disease outbreaks among people experiencing homelessness: a systematic review of prevention and mitigation strategies. Ann Epidemiol. 2022. doi:10.1016/j.annepidem.2022.03.004 35342013

[15] Umemura T , Mutoh Y , Kawamura T , et al. Efficacy of baloxavir marboxil on household transmission of influenza infection. J Pharm Heal Care Sci. 2020;6(1):21. doi:10.1186/s40780-020-00178-4 PMC7528271 33014405

[16] Ikematsu H , Hayden FG , Kawaguchi K , et al. Baloxavir marboxil for prophylaxis against influenza in household contacts. N Engl J Med. 2020;383(4):309‐320. doi:10.1056/nejmoa1915341 32640124

[17] *Influenza Update No. 421*; 2022.

[18] Gentilotti E , De Nardo P , Cremonini E , et al. Diagnostic accuracy of point‐of‐care tests in acute community‐acquired lower respiratory tract infections. A systematic review and meta‐analysis. Clin Microbiol Infect. 2022;28(1):13‐22. doi:10.1016/j.cmi.2021.09.025 34601148

[19] Klepser DG , Corn CE , Schmidt M , Dering‐Anderson AM , Klepser ME . Health care resource utilization and costs for influenza‐like illness among midwestern health plan members. J Manag Care Pharm. 2015;21(7):568‐573. doi:10.18553/jmcp.2015.21.7.568 PMC10398235 26108381

[20] Ku BS , Scott KC , Kertesz SG , Pitts SR . Factors associated with use of urban emergency departments by the U.S. homeless population. Public Health Rep. 2010;125(3):398‐405. doi:10.1177/003335491012500308 20433034 PMC2848264

[21] Poon LLM , Chan KH , Chu DKW , et al. Viral genetic sequence variations in pandemic H1N1/2009 and seasonal H3N2 influenza viruses within an individual, a household and a community. J Clin Virol. 2011;52(2):146‐150. doi:10.1016/j.jcv.2011.06.022 21802983 PMC3175291

[22] Chow EJ , Doyle JD , Uyeki TM . Influenza virus‐related critical illness: prevention, diagnosis, treatment. Crit Care. 2019;23(1):214. doi:10.1186/s13054-019-2491-9 31189475 PMC6563376

[23] Wille SM , Kemp KA , Greenfield BL , Walls ML . Barriers to healthcare for American Indians experiencing homelessness. J Soc Distress Homeless. 2017;26(1):1‐8. doi:10.1080/10530789.2016.1265211 29375241 PMC5783318

[24] Kryda AD , Compton MT . Mistrust of outreach workers and lack of confidence in available services among individuals who are chronically street homeless. Community Ment Health J. 2009;45(2):144‐150. doi:10.1007/s10597-008-9163-6 18807181

[25] McCosker LK , El‐Heneidy A , Seale H , Ware RS , Downes MJ . Strategies to improve vaccination rates in people who are homeless: a systematic review. Vaccine. 2022;40(23):3109‐3126. doi:10.1016/j.vaccine.2022.04.022 35484042 PMC9040475

[26] Uyeki TM , Hui DS , Zambon M , Wentworth DE , Monto AS . Influenza. Lancet. 2022;400(10353):693‐706. doi:10.1016/S0140-6736(22)00982-5 36030813 PMC9411419

