# Supplementary material for: Results from a test‐and‐treat study for influenza among residents of homeless shelters in King County, WA: A stepped‐wedge cluster‐randomized trial
Source: Influenza Other Respir Viruses. 2023 Jan 7;17(1):e13092. doi: 10.1111/irv.13092 (PMC9835442; doi:10.1111/irv.13092)
Supplement: Supplementary file 1 — Table S1. Shelter‐specific characteristics and ARI participant encounter numbers, 2019–2020 and 2020–2021 influenza seasons Table S2. Rapid on‐site molecular test results in comparison with RT‐PCR‐confirmed influenza test results Table S3. Influenza‐positive specimens with full genome sequences collected from shelter residents with <50% missing data. Figure S1. Weekly influenza virus detection by RT‐PCR, October 2019 – May 2021; includes nasal specimens collected from non‐ARI surveillance concurrently conducted at study site shelters Figure S2. Within‐subject change in viral load of specimen with detectable influenza RNA virus by RT‐PCR at study days 0, 2/3 and days 5/6/7 among those treated with an antiviral [file IRV-17-e13092-s001.docx]

**Appendix File**

***Study Power***

The study aimed to demonstrate a 50% reduction in symptomatic influenza virus infections after implementation of point-of-care molecular testing for influenza and early treatment with baloxavir or oseltamivir. Power calculations were based on an assumed 1.6% incidence rate per month, which was determined based on assumed 12% cumulative incidence rate during the pilot study’s 6-month influenza season and assuming 80% of cases are detected. The table below outlines the power based on various estimated effect sizes and number of shelters-seasons (shelters*season)

In all calculations we assumed a two-sided test with a = 0.05. Influenza seasons were assumed to last 6 months. In the general population, influenza incidence is expected to be 10%/season. We assumed incidence will be higher in the homeless shelter population and estimated incidence of 12%/season or, on average, about 2%/month. Shelters were each expected to contribute 200 person-months of observation and we assumed that we would detect 80% of the influenza cases at each shelter, so the detected incidence rate was estimated at 1.6%/month. These assumptions implied, in the absence of an intervention, approximately 3 detected cases of influenza per shelter per month (200*.02*.8).  Multiple seasons were considered independent replicates, equivalent to recruiting new shelters.

| **Power (%) to detect intervention effect as a function of RR and number of shelter-seasons. Assumptions: Six month influenza season, average shelter census = 200, effective control incidence rate = 1.6%/month (=2%*.8), SD(shelter) = .0028, SD(shelter*month) = .0032, SD(intervention) = 0.** | | | | | | | |
| --- | --- | --- | --- | --- | --- | --- | --- |
|  |  | **Number of shelter-seasons** | | | | | |
|  |  | **8** | **12** | **16** | **18** | **24** | **32** |
| **RR** | **.7** | 21 | 31 | 36 | 41 | 51 | 63 |
|  | **.65** | 27 | 40 | 48 | 54 | 65 | 77 |
|  | **.6** | 34 | 51 | 60 | 67 | 77 | 88 |
|  | **.5** | 52 | 72 | 81 | 86 | 93 | 98 |

Note: Simulations suggested a small loss of power if the incidence rate was not constant over the influenza season, but the loss was low (3-4%).

Assuming nine shelters participated for two seasons each (18 shelter-seasons), we had an estimated an 86 percent power to detect a risk ratio of 0.50 at a 0.05 two-sided significance level, assuming 200 participants per shelter. The incidence during intervention periods was compared to the incidence during non-intervention periods using general estimating equation models to control for clustering, differences in shelters, and time period.

***Antiviral Treatment***

Storage

Prepared kits of both study drugs were provided through Harborview Investigational Drug Services (IDS) and were kept locked safely and securely in either the administrative office or clinic room at the shelter site. Each had a coded kit number, with the first character designating the site/shelter, the second character designating the dose of the baloxavir or oseltamivir, and the rest of the numbers designating the specific kit number. All baloxavir tablets were kept on site for a period of two weeks and regularly reconciled with Harborview IDS.

Oseltamivir suspension was compounded with purified water containing 0.05% w/v sodium benzoate added as a preservative by Harborview IDS. Oseltamivir suspension kits included 10 disposable syringes for dosage, were stable for 10 days and kept in study- provided refrigerators at shelter sites. They were returned and reconciled to IDS from the shelters once the expiration date was < 5 days away. Temperature probes were kept on site to monitor and ensure the room and refrigerator temperatures did not surpass recommended levels.

Administration

Drug counseling was provided by all trained research staff. Participants were talked through any prohibited medications and contraindications. Baloxavir was provided as a directly observed therapy. For oseltamivir recipients, 10 capsules or a supply oseltamivir suspension were provided and instructed, to be taken by mouth, two times a day (morning and evening) for 5 days. For pediatric oseltamivir recipients (<12 years), a parent was provided a 5-day supply of oral suspension and instructed to keep the supply in the provided minifridge on-site with 24/7 access. Participants that received oseltamivir were advised that the treatment could be taken with or without food, but it was less likely to cause upset stomach if taken with food or milk. Crackers and juice were provided with oseltamivir treatment so that the first dose could be observed by the research staff. Participants that received oseltamivir were also advised to continue their treatment course until they finished the prescription, even if their symptoms began to improve.

***Genomic Sequencing***

Estimation of the number of sequenced cases due to intra-shelter transmission

We estimated the number of sequenced cases that were the result of intra-shelter transmission using the following approach: we identified groups of genomes from the same shelter that formed monophyletic clusters (to the exclusion of genomes from other shelters and from outside the study) with high bootstrap support (>95%). We then considered one genome in these groups to represent the index case while all others were considered to be the result of intra-shelter transmission. This approach provides a very conservative estimate for the number of cases due to intra-shelter transmission as groups of shelter genomes must be clearly distinct from other genomes to be considered an intra-shelter transmission group and because this method assumes that all cases in intra-shelter transmission groups have been identified by testing and have a corresponding sequenced virus, which is unlikely to be true.

Antiviral Resistance

We assessed the viral genomics generated for the study for the following mutations with reduced susceptibility to antivirals:

- Oseltamivir resistance mutations in NA
  - Influenza A(H1N1)pdm09 viruses: H275Y; I223R; Q136R,K ; V106K; T148A; D199T; I223T; I427T
  - Influenza B Victoria viruses: D197N, E; G104R; D432G; G145E; A200T; I221T; A245T; R270K
- Baloxavir resistance mutations in PA: I38L,M; E23G; A37T; E199G

***Sensitivity Analyses***

Asymptomatic / pauci-symptomatic influenza prevalence

*Methods:* Between 11/15/19 and 3/29/2020, residents that did not meet ARI criteria were eligible 1x/month for surveillance testing, regardless of whether their shelter was in the control or intervention period. This was done to capture cross-sectional prevalence of asymptomatic (no symptoms) and paucisymptomatic (<2 ARI symptoms and no acute cough) influenza virus infections. From 3/30/20 onwards, asymptomatic and paucisymptomatic surveillance testing was available every day that shelter kiosks were staffed; participation was limited to once/week. Participants self-collected nasal samples from 3/6/20 onwards with kiosk staff supervision.

*Statistical Analysis:* The prevalence of asymptomatic and pauci-symptomatic influenza-positive samples was also assessed as an endpoint. To calculate this measure, we included the monthly participation of non-ARI eligible subjects prior to 3/30/2020 and those routinely tested following this date once standard surveillance was expanded in our denominator.

*Results:* Over the entire study period, 8 influenza virus infections were identified among 5,567 non-ARI participant encounters; infection prevalence among non-ARI encounters was 0.14% compared to 4.0% among ARI encounters (**Figure 2b**; **Supplemental Figure 1**).

Agreement between rapid molecular testing and RT-PCR testing

*Statistical Analysis:* Restricting the analysis to all participant encounters/specimens collected that had an on-site rapid molecular test performed, the sensitivity, specificity, and Cohen’s kappa coefficient (κ) were calculated; concordance measures compared the influenza rapid on-site test with the TaqMan assay, where the TaqMan assay represented the gold standard. A p-value <0.05 was considered statistically significant.

*Results:* Of the 21 rapid molecular test positives, 19 also had influenza virus detected by RT-PCR (**Supplemental** **Table 2**). Measures of agreement of the rapid molecular test were higher for influenza A than B: sensitivity and specificity were both 100% for influenza A (κ = 1). For influenza B, sensitivity was 88.9%, specificity was 99.2%, and Cohen’s kappa was 0.881. The overall sensitivity of the on-site Abbott rapid molecular test was 90.5% and the specificity was 99.6%, suggesting on-site test performance was concordant with RT-PCR (κ = 0.901)

Proportion of samples with detectable influenza RNA virus at days 2/3 and days 5/6/7

*Statistical Analysis:* Participants that received an antiviral that did not become lost to follow-up had nasal specimens assessed for continued detectable influenza virus. Influenza viral RNA levels using an OpenArray relative cycle threshold (CRT) values were measured for each influenza-positive specimen collected prior to and following antiviral treatment. Mean CRT values and corresponding standard deviations (SD) were calculated. CRT is a relative cycle threshold and an alternative to Ct (cycle threshold) that is used specifically by Thermo’s OpenArray platform. CRT is calculated based on the amplification curve alone, versus Ct which considers all the curves for a specific target to determine the threshold. These two measures are strongly correlated and therefore can be interpreted the same way.

*Results:* Among the 14 test-and-treat intervention participants that returned for their first follow-up study visit (2-3 days post-treatment) and provided a nasal specimen, 7 (50%) had detectable influenza virus. One participant who returned for the second and final visit had no virus detected in their specimen (CRT = 0); all missing swabs’ CRT values were also treated as zero (**Supplemental** **Figure 2**). The mean CRT value at the first follow-up visit was 19.9 (SD: 5.3); by comparison, the mean value at treatment was 17.2 (SD: 5.1).

***Supplemental Table 1. Shelter-specific characteristics and ARI participant encounter numbers, 2019-2020 and 2020-2021 influenza seasons***

|  | **Shelters*****, 2019-2020 influenza season (N=1,159)** | | | | | | | | | | | | | |
| --- | --- | --- | --- | --- | --- | --- | --- | --- | --- | --- | --- | --- | --- | --- |
| **Variable** | A | B | C | D | E | F | G | H | I | J | K | L | M | O |
| Age – median (IQR) | 40.0 [18.8] | 45.0 [10.0] | 22.0 [4.00] | 25.0 [25.8] | 19.0 [28.0] | 47.5 [21.3] | 50.5 [18.3] | 6.00 [29.3] |  |  |  | 46.0 [16.0] | 56.0 [7.00] | 14.0 [20.0] |
| Duration of homelessness |  |  |  |  |  |  |  |  |  |  |  |  |  |  |
| *<6 months* | 18 (19.6%) | 36 (27.9%) | 16 (24.6%) | 45 (59.2%) | 50 (61.0%) | 61 (48.0%) | 10 (37.0%) | 2 (25.0%) |  |  |  | 104 (35.4%) | 16 (8.04%) | 18 (60.0%) |
| *6 – 12 months* | 30 (32.6%) | 21 (16.3%) | 10 (15.4%) | 14 (18.4%) | 19 (23.2%) | 16 (12.6%) | 4 (14.8%) | 2 (25.0%) |  |  |  | 42 (14.3%) | 20 (10.1%) | 4 (13.3%) |
| *13 – 24 months* | 11 (12.0%) | 26 (20.2%) | 5 (7.69%) | 10 (13.2%) | 4 (4.88%) | 10 (7.87%) | 7 (25.9%) | 3 (37.5%) |  |  |  | 31 (10.5%) | 25 (12.6%) | 1 (3.33%) |
| *> 24 months* | 33 (35.9%) | 46 (35.7%) | 34 (52.3%) | 7 (9.21%) | 9 (11.0%) | 40 (31.5%) | 6 (22.2%) | 1 (12.5%) |  |  |  | 117 (39.8%) | 138 (69.3%) | 7 (23.3%) |
| Sex |  |  |  |  |  |  |  |  |  |  |  |  |  |  |
| *Male* | 0 (0%) | 117 (90.7%) | 40 (64.5%) | 22 (28.2%) | 34 (41.5%) | 131 (99.2%) | 18 (64.3%) | 1 (12.5%) |  |  |  | 203 (68.1%) | 213 (100%) | 9 (29.0%) |
| *Female* | 91 (100%) | 12 (9.30%) | 22 (35.5%) | 56 (71.8%) | 48 (58.5%) | 1 (0.758%) | 10 (35.7%) | 7 (87.5%) |  |  |  | 95 (31.9%) | 0 (0%) | 22 (71.0%) |
| Maximum capacity | 60 | 100 | 45 | 185 | 70 | 60 | 275 | 275 |  |  |  | 200 | 212 | 100 |
| No. of enrollments |  |  |  |  |  |  |  |  |  |  |  |  |  |  |
| *Control period* | 46 (50.0%) | 34 (26.2%) | 31 (46.3%) | 59 (75.6%) | 24 (29.3%) | 9 (6.82%) | 28 (100%) | 8 (100%) |  |  |  | 234 (78.5%) | 5 (2.35%) | 1 (3.23%) |
| *Intervention period* | 46 (50.0%) | 96 (73.8%) | 36 (53.7%) | 19 (24.4%) | 58 (70.7%) | 123 (93.2%) | 0 (0%) | 0 (0%) |  |  |  | 64 (21.5%) | 208 (97.7%) | 30 (96.8%) |
|  | **Shelters, 2020-2021 influenza season (N= 124)** | | | | | | | | | | | | | |
| **Variable** | A | B | C | D | E | F | G | H | I | J | K | L | M | O |
| Age – median (IQR) | 52.0 [4.50] | 42.0 [14.0] | 22.5 [2.75] | 19.0 [12.0] | 5.50 [21.5] | 53.0 [18.8] | 49.0 [10.5] | 11.0 [36.0] | 54.0 [6.50] | 53.0 [0.750] | 46.5 [8.25] |  |  |  |
| Duration of homelessness |  |  |  |  |  |  |  |  |  |  |  |  |  |  |
| *<6 months* | 2 (66.7%) | 0 (0%) | 2 (20.0%) | 4 (80.0%) | 2 (50.0%) | 1 (14.3%) | 5 (9.26%) | 4 (80.0%) | 0 (0%) | 0 (0%) | 2 (25.0%) |  |  |  |
| *6 – 12 months* | 1 (33.3%) | 0 (0%) | 1 (10.0%) | 1 (20.0%) | 0 (0%) | 3 (42.9%) | 12 (22.2%) | 1 (20.0%) | 1 (9.09%) | 3 (50.0%) | 3 (37.5%) |  |  |  |
| *13 – 24 months* | 0 (0%) | 0 (0%) | 3 (30.0%) | 0 (0%) | 0 (0%) | 1 (14.3%) | 10 (18.5%) | 0 (0%) | 0 (0%) | 0 (0%) | 1 (12.5%) |  |  |  |
| *> 24 months* | 0 (0%) | 5 (100%) | 4 (40.0%) | 0 (0%) | 2 (50.0%) | 2 (28.6%) | 27 (50.0%) | 0 (0%) | 10 (90.9%) | 3 (50.0%) | 2 (25.0%) |  |  |  |
| Sex |  |  |  |  |  |  |  |  |  |  |  |  |  |  |
| *Male* | 0 (0%) | 6 (100%) | 5 (71.4%) | 1 (20.0%) | 4 (66.7%) | 7 (100%) | 40 (72.7%) | 3 (60.0%) | 10 (100%) | 6 (100%) | 8 (100%) |  |  |  |
| *Female* | 3 (100%) | 0 (0%) | 2 (28.6%) | 4 (80.0%) | 2 (33.3%) | 0 (0%) | 15 (27.3%) | 2 (40.0%) | 0 (0%) | 0 (0%) | 0 (0%) |  |  |  |
| Maximum capacity | 60 | 100 | 45 | 185 | 70 | 60 | 275 | 275 | 45 | 34 |  |  |  |  |
| No. of enrollments |  |  |  |  |  |  |  |  |  |  |  |  |  |  |
| *Control period* | 3 (100%) | 0 (0%) | 7 (70.0%) | 3 (60.0%) | 1 (16.7%) | 7 (87.5%) | 4 (7.27%) | 0 (0%) | 3 (27.3%) | 5 (83.3%) | 2 (25.0%) |  |  |  |
| *Intervention period* | 0 (0%) | 7 (100%) | 3 (30.0%) | 2 (40.0%) | 5 (83.3%) | 1 (12.5%) | 51 (92.7%) | 5 (100%) | 8 (72.7%) | 1 (16.7%) | 6 (75.0%) |  |  |  |

******* *During the summer surveillance period: Shelter K replaced B; J replaced F; G replaced L; H replaced O; I replaced M*

***Supplemental Table 2. Rapid on-site molecular test results in comparison with RT-PCR-confirmed influenza test results***

| Part A. 2x2 Table of Abbott Test compared to TaqMan Assay for Influenza A | | | |
| --- | --- | --- | --- |
|  | *RT-PCR-confirmed influenza A positive* | *RT-PCR-confirmed influenza A negative* | *Total* |
| *Abbott-positive influenza A* | 3 | 0 | 3 |
| *Abbott-negative influenza A* | 0 | 266 | 266 |
| *Total* | 3 | 266 | 269 |
| Part B. 2x2 Table of Abbott Test compared to TaqMan Assay for Influenza B | | | |
|  | *RT-PCR-confirmed influenza B positive* | *RT-PCR-confirmed influenza B negative* | *Total* |
| *Abbott-positive influenza B* | 16 | 2 | 18 |
| *Abbott-negative influenza B* | 2 | 249 | 251 |
| *Total* | 18 | 251 | 269 |
| Part C. 2x2 Measures of Agreement | | | |
|  | *Influenza A* | *Influenza B* | *Overall* |
| *Sensitivity (95% CI)* | 100.0% (29.2, 100.0) | 88.9% (65.3, 98.6) | 90.5% (69.6, 98.8) |
| *Specificity (95% CI)* | 100.0% (98.6, 100.0) | 99.2% (97.2, 99.9) | 99.6% (98.6, 99.95) |
| $\boldsymbol{\kappa}$ *(95% CI)* | 1 | 0.881 (0.765, 0.997) | 0.901 (0.804, 0.998) |

***Supplemental Table 3. Influenza-positive specimens with full genome sequences collected from shelter residents with <50% missing data.***

| Influenza A | | |
| --- | --- | --- |
| Shelter (N=7) | Type | Date |
| L (n=3) | Mixed gender adults | 2020-02-01  2020-02-01  2020-02-05  2020-02-11 |
| D (n=2) | Family | 2020-02-01  2020-02-01 |
| F (n=1) | 381021 | 2020-01-29 |
| **Influenza B** | | |
| Shelter (N=18) | Type | Dates |
| E (n=9) | Family | 2019-12-26  2019-12-27  2019-12-27  2019-12-27  2019-12-27  2019-12-27  2019-12-28  2019-12-28  2019-12-28 |
| O (n=5) | Family | 2019-12-04  2019-12-05  2019-12-06  2020-01-10  2020-01-22 |
| L (n=1) | Mixed gender adults | 2020-01-17  2020-01-25 |
| D (n=1) | Family | 2019-12-27 |
| C (n=1) | Young adult | 2020-02-01 |

***Supplemental Figure 1. Weekly influenza virus detection by RT-PCR, October 2019 – May 2021; includes nasal specimens collected from non-ARI surveillance concurrently conducted at study site shelters***

***
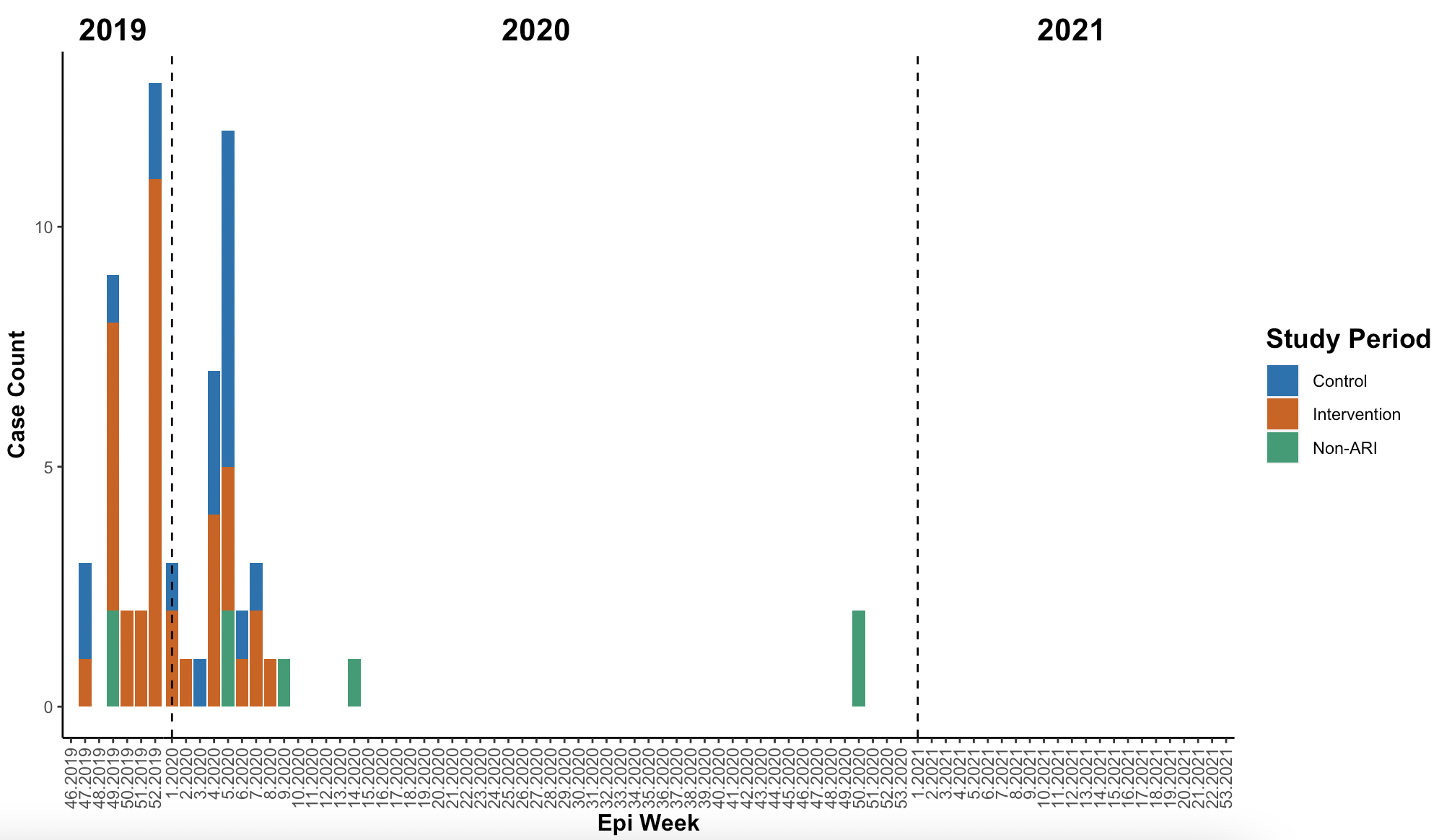
***

***Supplemental Figure 2. Within-subject change in viral load of specimen with detectable influenza RNA virus by RT-PCR at study days 0, 2/3 and days 5/6/7 among those treated with an antiviral***

*
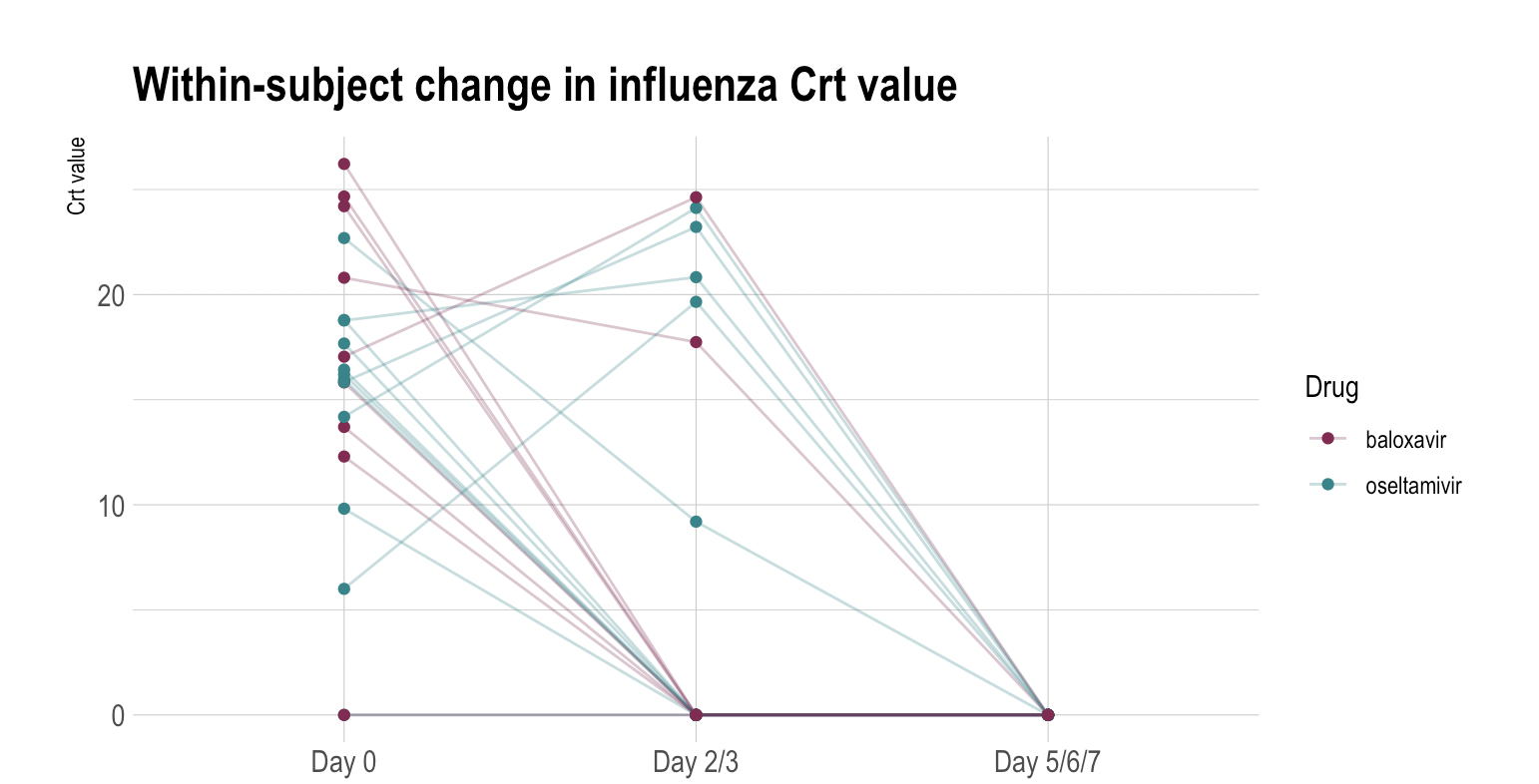
*
